# An explainable artificial intelligence framework for clinical decision support in stroke discharge planning

**DOI:** 10.1371/journal.pone.0353683

**Published:** 2026-07-15

**Authors:** Seifollah Gholampour, Arshia Dehghan, Evelyn B. Voura, Jesse Huang, Mohammad Jamshidi, George W. Koutsouras

**Affiliations:** 1 Department of Neurological Surgery, University of Chicago Medicine, Chicago, Illinois, United States of America; 2 Crouse Neuroscience Institute, Crouse Health at Crouse Hospital, Crouse Medical Practice, Syracuse, New York, United States of America; 3 Department of Neuroscience and Physiology, State University of New York (SUNY) Upstate Medical University, Syracuse, New York, United States of America; 4 Department of Neurology, Northwestern University, Chicago, Illinois, United States of America; 5 Department of Neurosurgery, Upstate University Hospital, Syracuse, New York, United States of America; University of Toronto, CANADA

## Abstract

**Background:**

Stroke, a leading cause of global mortality and disability, requires accurate prediction of discharge outcomes to support early care planning. We developed an explainable artificial intelligence (AI) framework to predict four discharge categories (home, specialized care, home with help, expired) and identify key predictors.

**Methods:**

This single-center retrospective study included 1,731 patients with ischemic stroke, hemorrhagic stroke, or transient ischemic attack (TIA). Twenty routinely available electronic health record variables were used. Ten classifiers were compared using stratified 5-fold cross-validation, and the final model was calibrated with training-set out-of-fold predictions and interpreted using SHapley Additive exPlanations (SHAP).

**Results:**

The multilayer perceptron (MLP) achieved the highest mean cross-validated macro-F1 score and was selected as the best-performing model. On the independent hold-out test set, the MLP achieved an accuracy of 0.646, macro-specificity of 0.873, macro-precision of 0.559, macro-sensitivity of 0.557, and macro-F1 of 0.548. Class-wise area under the receiver operating characteristic curve (AUC) values were 0.901 for home, 0.874 for specialized care, 0.674 for home with help, and 0.889 for expired. SHAP analysis identified admission National Institutes of Health Stroke Scale (NIHSS), length of stay, age, and primary diagnosis as shared predictors across all discharge categories. The SHAP age-threshold analysis identified 72.0 years as a clinically relevant threshold associated with a lower likelihood of home discharge and higher likelihoods of specialized care, home with help, and expired discharge status. The model also highlighted clinically actionable or addressable domains, including blood glucose, depression, insurance type, last known well time, anticoagulant use, and treatment-related variables.

**Conclusion:**

This interpretable AI-based framework identified clinically relevant predictors of stroke discharge disposition within this single-center retrospective dataset. These findings may inform future decision-support development; however, clinical implementation, resource optimization, and health-system impact require prospective multicenter validation.

## Introduction

Stroke persists as a leading global cause of mortality, ranking as the fifth-leading cause of death in the United States, with a median prevalence of 3.4% among U.S. adults in 2022 [1]. Acute stroke is a heterogeneous condition rather than a single uniform disease entity. In clinical studies and prognostic modeling, differentiating hemorrhagic stroke from ischemic stroke and its etiologic subtypes—including cardioembolic stroke, lacunar infarction, atherothrombotic infarction, infarction of unusual etiology, and infarction of undetermined etiology—is important because these entities may differ substantially in pathophysiology, risk-factor distribution, stroke severity, prognosis, and clinical outcomes [2]. Beyond its high mortality burden, stroke frequently results in impairments requiring comprehensive post-acute care planning [3], including discharge to home, specialized rehabilitation centers, or home with help [4]. The determination of discharge disposition, reflecting the transition to various care settings, is indicative of the severity of neurological injury, the trajectory of functional recovery, and the overall prognosis [5]. While the early prediction of discharge disposition is vital for guiding immediate care transitions, determining rehabilitation intensity, and optimizing resource allocation [4,6,7], it is important to acknowledge that discharge is not a static endpoint. Rather, the chosen discharge destination significantly influences a patient’s subsequent recovery trajectory by shaping their access to and the intensity of post-discharge rehabilitation. Suboptimal discharge dispositions increase healthcare costs, reduce quality of life, and elevate 30-day readmission rates [8,9], underscoring the health systems imperative to mitigate stroke’s considerable economic burden¹ ($417.9 billion annually in the US for cardiovascular diseases [1]) through predictive modeling of discharge outcomes, particularly anticipating mortality and the need for specialized care. Therefore, early and accurate prediction of discharge status is paramount not only for optimizing clinical decision-making but also for enhancing system-wide efficiency.

Prior studies have used conventional statistical approaches to identify variables associated with discharge outcomes [10,11]. Traditional regression models can accommodate nonlinearities and interactions when these are prespecified [12,13], and modern penalized methods can also capture substantial model complexity [14,15]. However, in clinically complex datasets, such as discharge disposition prediction in acute stroke, where such relationships may not be known in advance, manual specification of functional forms and candidate interactions can be challenging and may miss important patterns if relevant nonlinear terms or interactions are not explicitly specified. In this setting, the advantage of modern machine learning methods lies in their ability to search a broader hypothesis space for potentially complex nonlinearities and interactions with less reliance on manual feature engineering and prespecification. This challenge is well illustrated by prior findings. The role of tissue plasminogen activator (tPA) administration in improving discharge outcomes remains debated, with some studies reporting conflicting results depending on stroke subtypes and cohort characteristics [16,17]. Similar inconsistencies have also been observed regarding the effects of age [11,17–19] and sex [18,20]. While recent advances in artificial intelligence (AI) have begun addressing some of these methodological constraints, existing models remain narrowly focused on predicting functional outcomes—such as modified Rankin Scale (mRS) scores [12,21], NIH Stroke Scale (NIHSS) trajectories [22], Barthel Index (BI) values [23] or binary risks (e.g., mortality) [24] at discharge or follow-up intervals—rather than actionable discharge destinations. Although functional outcome scores are important for assessing acute stroke severity, they may not fully capture recovery potential, rehabilitation capacity, or differences in discharge trajectory among patients with similar initial assessments. This limitation underscores the necessity for AI prognostication to provide actionable insights for clinicians determining post-acute care pathways (home, specialized care, home with help, or mortality) to improve care quality, resource allocation, and patient-centered planning. On the other hand, even prior AI studies predicting discharge disposition show functional scores often failing as key determinants of destination categories [25], reinforcing the need for models directly targeting these outcomes.

This study aims to develop an explainable AI framework to identify patients’ immediate post-acute care needs upon hospital discharge (home, specialized care, home with help, expired), facilitating early mobilization of resources. Unlike prior AI works that prioritize secondary functional outcomes or binary risks (e.g., mortality), our model directly addresses the decision point central to post-acute care coordination: determining the *destination* of care transitions, not merely the *degree* of functional recovery. By integrating SHapley Additive exPlanations (SHAP), we also systematically quantify variable contributions across all potential interactions, identifying key predictors [26,27]. This approach may equip clinicians with actionable insights to optimize care pathways, align resources, and mitigate risks, thereby advancing patient-centered outcomes and system-wide efficiency.

## Methods

All procedures were in accordance with the ethical standards of the responsible committee on human experimentation and with the Helsinki Declaration of 1964 and its later amendments. The study protocol was approved by the Crouse Hospital Institutional Review Board (IRB; #2021.0125) on 15/02/2021. Due to the retrospective nature of the study, the requirement for individual patient consent was waived by the IRB. All patient data were fully anonymized prior to analysis to ensure confidentiality. Data for this research were accessed between 01/03/2021 and 31/05/2021. No animal studies were performed. Furthermore, this study was reported in accordance with the TRIPOD+AI statement, and the completed TRIPOD+AI checklist is provided as S1 Checklist.

### Data collection

This single-center retrospective study at Crouse Hospital (Syracuse, NY) collected data from electronic health records (EHR) for patients diagnosed with stroke (ischemic, hemorrhagic) or transient ischemic attack (TIA) who presented to the emergency department (ED) between January 2019 and January 2021. Patient management adhered to the hospital’s clinical protocol for suspected stroke (S1 Fig), which prioritizes timely interventions—including CT imaging, thrombolytic eligibility assessment, and reperfusion therapies. Twenty variables spanning demographic characteristics, clinical presentation, vital signs, laboratory measurements, and neuropsychiatric/substance use indicators were collected for each patient and categorized as outlined in Table 1. These 20 predictors were pre-specified a priori based on clinical relevance, routine availability in the electronic health record during acute stroke evaluation, and consensus among the clinical investigators involved in the study. No separate data-driven feature selection approach was applied before model development, and all pre-specified predictors were retained in the analyses. Discharge disposition was classified into four categories: home, specialized care, home with help, and expired.

**Table 1:** Overview of variables used and bivariate analysis. P-values 1, 2, and 3 correspond to comparisons of home vs. specialized care, home vs. home with help, and home vs. expired, respectively. The units for LDL, systolic blood pressure, and blood glucose are mg/dL, mmHg, and mg/dL, respectively.

Categorical variables						
Variable	Category	Count (%)	P-value 1	P-value 2	P-value 3	Missing Count (%)
Sex	Female	892 (51.5%)	**<0.001**	**<0.001**	0.093	0 (0.0%)
	Male	839 (48.5%)				
Last Known Well	< 3 hours	634 (36.8%)	**<0.001**	0.067	**0.019**	7 (0.4%)
	3 to 6 hours	197 (11.4%)				
	6 to 12 hours	199 (11.5%)				
	12-24 hours	269 (15.6%)				
	> 24 hours	425 (24.7%)				
Smoking Status	Never	855 (50.4%)	**0.032**	0.337	**0.010**	35 (2.0%)
	Former	527 (31.1%)				
	Current	314 (18.5%)				
Aspirin Use	No	1067 (61.6%)	0.912	0.114	0.585	0 (0.0%)
	Yes	664 (38.4%)				
Anticoagulant	No	1244 (71.9%)	0.664	**0.024**	**<0.001**	0 (0.0%)
	Yes	487 (28.1%)				
tPA administered	No	1557 (89.9%)	**0.004**	0.054	**<0.001**	0 (0.0%)
	Yes – No bleed	157 (9.1%)				
	Yes – With bleed	17 (1.0%)				
Drug Screen Positive	No	1629 (94.1%)	0.322	0.756	0.221	0 (0.0%)
	Yes	102 (5.9%)				
Primary Diagnosis	Ischemic	1263 (73.0%)	**<0.001**	**<0.001**	**<0.001**	0 (0.0%)
	TIA	314 (18.1%)				
	Hemorrhage	154 (8.9%)				
Race	White	1514 (87.5%)	0.171	0.601	0.287	0 (0.0%)
	Black	172 (9.9%)				
	Hispanic	23 (1.3%)				
	Asian	8 (0.5%)				
	Other	14 (0.8%)				
Previous Stroke	No	1262 (72.9%)	0.454	**0.008**	**0.021**	0 (0.0%)
	Yes	469 (27.1%)				
Depression	No	1339 (77.4%)	0.238	**0.018**	0.824	0 (0.0%)
	Yes	392 (22.6%)				
Insurance Type	Commercial	318 (18.4%)	**<0.001**	**<0.001**	**<0.001**	0 (0.0%)
	Medicare	1177 (68.0%)				
	Medicaid	133 (7.7%)				
	Other	103 (6.0%)				
Service	No	712 (41.1%)	**0.011**	0.637	0.383	0 (0.0%)
	Yes	1019 (58.9%)				
Troponin	Normal	1143 (72.4%)	**0.031**	0.793	**<0.001**	152 (8.8%)
	High	436 (27.6%)				
**Numerical variables**						
LOS (Days)	Continuous	4.24 ± 4.36	**<0.001**	**<0.001**	**<0.001**	0 (0.0%)
Age (Years)	Continuous	72.08 ± 13.84	**<0.001**	**<0.001**	**<0.001**	0 (0.0%)
LDL	Continuous	98.90 ± 43.67	0.083	0.198	**0.022**	167 (9.6%)
Systolic BP	Continuous	150.08 ± 25.14	0.190	0.105	**0.006**	3 (0.2%)
Blood Glucose	Continuous	138.31 ± 74.24	**<0.001**	**<0.001**	**<0.001**	13 (0.8%)
Admission NIHSS	Continuous	4.88 ± 6.43	**<0.001**	**<0.001**	**<0.001**	82 (4.7%)

LDL: Low-density lipoprotein; tPA: tissue plasminogen activator; NIHSS: NIH stroke scale; SD: Standard deviation.

### Operational definition and timing of predictor capture

Predictor variables were defined using structured EHR data available during the acute hospitalization. Specifically: (1) laboratory values and vital signs reflected the earliest available measurements recorded immediately upon ED triage; (2) admission NIHSS represented the initial neurological assessment scored by the clinical team before treatment initiation; (3) depression and other comorbidities were defined based on prior documented medical history or the active problem list available in the EHR at the time of admission and were not inferred solely from medication proxies or unstructured note abstraction; and (4) last known well (LKW) time was extracted from ED stroke documentation and categorized as <3 hours, 3–6 hours, 6–12 hours, 12–24 hours, and >24 hours. Cases with undocumented LKW times (Table 1: n = 7, 0.4%) were treated as missing and handled using multiple imputation by chained equations (MICE), as detailed below.

### Missing data handling

Missing data were present in seven variables, ranging from 0.4% to 9.6%, as shown in Table 1. Missing values were handled using a mixed-type MICE-style iterative imputation procedure implemented with IterativeImputer. The imputation procedure was incorporated within the modeling pipeline to reduce information leakage. Thirty imputation iterations were used. This value was selected as a conservative upper limit for the chained-equation iterations, given the relatively low proportion of missingness in the dataset, and to allow sufficient stabilization of the imputation process before downstream model training and evaluation.

### Exploratory principal component analysis

To provide an exploratory visualization of the overall structure of the dataset, we performed a three-dimensional principal component analysis (PCA) using all 20 clinical variables. PCA was used solely for descriptive visualization of the degree of overlap and relative organization of discharge categories in reduced-dimensional space and was not used for feature selection, model training, or model evaluation.

### Bivariate analysis

We conducted bivariate analyses to assess baseline associations between discharge outcomes, using “home” as the reference category. Chi-square tests evaluated relationships between categorical features and discharge categories, substituting Fisher’s exact test when cell frequencies were below five. Numerical variables were initially tested for normality using Shapiro-Wilk tests, and comparisons across discharge groups were performed with one-way analysis of variance (ANOVA), for normally distributed data or Kruskal-Wallis tests, for non-normal data.

### Artificial intelligence analysis

We employed AI-based predictive modeling, defining the target variable (discharge disposition) as a multiclass outcome with four distinct categories: home (class 0), specialized care (class 1), home with help (class 2), and expired (class 3). The predictive features included 20 variables related to demographic information, clinical presentation, treatment, vital signs, laboratory measurements, and neuropsychiatric/substance use factors, as outlined in Table 1. The dataset was randomly partitioned into training (80%) and testing (20%) subsets. Ten classifiers were utilized to predict discharge status: logistic regression, support vector classifier (SVC), decision tree, k-nearest neighbors (KNN), random forest, adaptive boosting, extreme gradient boosting (XGBoost), categorical boosting, a stacked ensemble (random forest + XGBoost), and multilayer perceptron (MLP). A stacked ensemble combining random forest and XGBoost was included as an exploratory hybrid benchmark because these two tree-based methods offer complementary strengths for structured clinical data. Random forest is variance-reducing and robust through bagging and random feature selection, whereas XGBoost can reduce bias and capture complex nonlinear relationships through boosting. The ensemble was implemented using a logistic regression meta-learner trained on the predicted probabilities of the two base models after individual hyperparameter tuning.

### Multiclass AI model development, tuning, and evaluation

All classifiers were trained using their standard multiclass implementations for the four-class discharge outcome. Most classifiers directly support multiclass classification, whereas scikit-learn’s SVC uses a native one-vs-one (OvO) strategy for multiclass training. We did not impose a common one-vs-rest training wrapper across all models. For class-specific performance evaluation, including receiver operating characteristic (ROC) curve / area under the ROC curve (AUC) and calibration analyses, a one-vs-rest (OvR) framework was used for each discharge category. This approach provides clinically interpretable, destination-specific estimates by comparing each outcome against all remaining outcomes. For the SVC, the OvR evaluation was performed using the final class-probability outputs and did not alter the underlying one-vs-one training strategy.

The final selected MLP classifier was implemented using scikit-learn. The tuned network consisted of a single hidden layer with 48 neurons and a logistic activation function, with an output layer generating class probabilities for the four discharge disposition categories. The model was trained using the Adam optimizer with a constant learning rate of 0.0007, mini-batch size of 8, and a maximum of 3000 training iterations. No dropout or batch normalization layers were used. Regularization was instead performed using L2 weight decay, with a final selected alpha value of 0.03, together with early stopping. Early stopping was enabled with a tolerance of 1 × 10^-4 and n_iter_no_change = 35. For early stopping, an internal validation fraction of 0.25 was used. During cross-validation, this internal validation subset was drawn only from the training portion of each fold. No independent hold-out test data were used for early stopping, hyperparameter tuning, model selection, probability calibration, or threshold determination.

Given the class imbalance—home (44.9%), specialized care (29.5%), home with help (18.3%), and expired (7.3%)—we incorporated class-imbalance handling into model training. Native class weighting was used when supported, whereas weighted-resampling fallback approximated class-weighted learning for the MLP. No synthetic oversampling method, including synthetic minority over-sampling technique (SMOTE), was applied [28]. Hyperparameter tuning was performed exclusively within the training set using stratified 5-fold cross-validation. Multiple performance metrics were evaluated during cross-validation; however, the primary selection and refit metric was the mean cross-validated macro-F1 score, which was selected a priori because the outcome was multiclass and class-imbalanced. The best-performing classifier was selected based on the highest mean cross-validated macro-F1 score across the validation folds. Accuracy, AUROC, sensitivity, and specificity were additionally examined as secondary performance measures but did not alter the predefined model-selection criterion. The full hyperparameter search spaces and final selected settings for all classifiers are provided in S1 Table. After hyperparameter selection, probability calibration of the final selected MLP classifier was performed using vector scaling fitted on out-of-fold predictions from the training set only. Class-specific threshold tuning was also performed using calibrated out-of-fold predictions from the training set only. The independent hold-out test set was strictly isolated from hyperparameter tuning, model selection, probability calibration, and threshold determination. The hold-out test set was used only after final model selection for final performance evaluation and post hoc SHAP interpretation. Calibration performance was assessed on the independent hold-out test set using class-wise one-vs-rest reliability diagrams. For each discharge category, calibration performance was summarized using the Brier score and expected calibration error (ECE). Ten bins were used for each class-wise calibration curve. Learning curve analysis was performed to evaluate model stability by plotting training and cross-validation macro-F1 scores across increasing training-set sizes. Generalization was further validated by comparing cross-validation metrics to hold-out test performance, ensuring minimal divergence.

### SHAP analysis

To transparently identify key predictors of discharge disposition and ensure interpretability, we conducted a systematic SHAP analysis on the best-performing classifier. The process comprised three rigorous steps [27]:

***- Step 1:*** Feature Ranking and Subset Aggregation:
  *Class-Specific SHAP Importance:* For each discharge class (home, specialized care, home with help, expired), features were ranked by descending SHAP importance values. SHAP importance reflects the average absolute impact of a feature on model predictions across all instances.
  *Threshold-Based Subsets:* From each ranked list, the top 65%, 70%, 75%, 80%, and 85 of features were iteratively selected. Subsets from all four classes were then aggregated into a single union set, ensuring comprehensive coverage of predictors relevant to all discharge outcomes.
***- Step 2:*** Iterative Model Retraining and Threshold Selection
  *Retraining on Subsets:* The best-performing model (with original hyperparameters) was retrained on each aggregated subset (65%, 70%,  ..., 85%).
  *Performance Evaluation:* The performance of the classifier, trained on each specific subset of features using the original hyperparameters, was compared to that of the full-feature model using accuracy, macro-averaged F1-score, and AUC to identify the optimal feature subset.
***- Step 3:*** Sensitivity Analysis for Robustness Validation:
  To validate stability, features were individually added to or removed from the optimal subset. A subset was considered robust if perturbation (adding or removing any single feature) resulted in minimal alteration in accuracy, F1-score, and AUC values, with values remaining close to those of the original full-featured model.

The supplementary analysis code and serialized model-related artifacts are provided as S1 Code.

## Results

**Fig 1:**
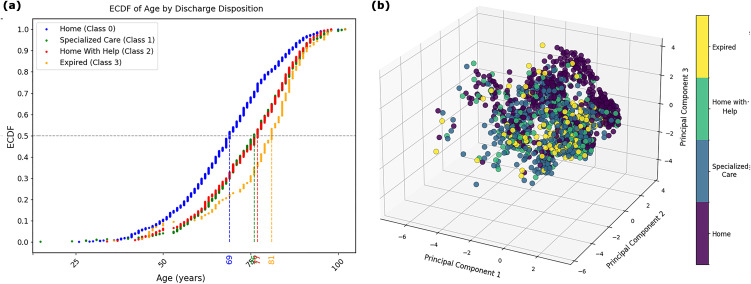
Empirical cumulative distribution function (ECDF) and Principal component analysis (PCA) of Discharge disposition. (a) ECDF of age stratified by discharge disposition, showing the distribution of patient age across the four discharge categories: home (class 0, blue), specialized care (class 1, green), home with help (class 2, red), and expired (class 3, orange). Vertical dashed lines mark age thresholds where 50% of patients in each category fall below. (b) Exploratory PCA visualization of discharge categories in reduced-dimensional space, showing partial organization of classes with substantial overlap across outcome groups.

The study cohort comprised 1,731 patients (mean age 72.1 ± 13.8 years; Fig 1a). Discharge dispositions were categorized as home (n = 778, 44.9%), specialized care (n = 510, 29.5%), home with help (n = 317, 18.3%), and expired (n = 126, 7.3%). A three-dimensional exploratory PCA was performed to visualize the global structure of the dataset in a reduced-dimensional linear space (Fig 1b). Each point represents an individual patient, and colors indicate the four discharge disposition categories. The PCA plot demonstrated substantial overlap among the home, specialized care, home with help, and expired groups, with no clearly separated class-specific clusters. Although partial organization of the outcome categories was observed, the marked overlap suggested that discharge disposition groups were not fully separable using the leading linear components alone. This finding supported the use of supervised classification models capable of capturing more complex, potentially nonlinear relationships and multivariable patterns among routinely available clinical predictors.

### Statistical analysis

Bivariate analysis revealed numerous significant associations between baseline variables and discharge disposition when compared to the reference category (discharge to home). Specifically, 12 predictors were associated with discharge to specialized care, 10 with discharge to home with help, and 14 with in-hospital mortality (expired) (Table 1). While informative for initial assessment, bivariate analyses do not account for the complex interdependencies between variables.

### Explainable AI analysis

**Fig 2:**
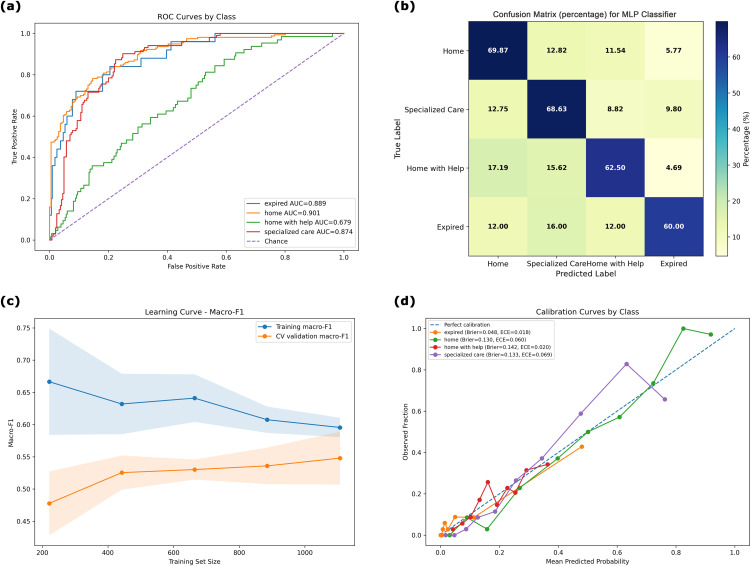
Performance evaluation of the final MLP classifier. (a) Class-wise one-vs-rest ROC curves with AUC values for home, specialized care, home with help, and expired discharge outcomes. (b) Percentage-based confusion matrix showing predicted versus true discharge categories. (c) Learning curve showing training and cross-validation macro-F1 across increasing training-set sizes. (d) Class-wise one-vs-rest calibration curves for the MLP classifier, with Brier scores and ECE values indicating agreement between predicted probabilities and observed outcome frequencies.

According to the stratified 5-fold cross-validation results on the training set, the MLP classifier achieved the highest mean cross-validated macro-F1 score among the ten evaluated classifiers and was therefore selected as the best-performing model for subsequent calibration and SHAP analyses. On the independent hold-out test set, the selected MLP achieved an accuracy of 0.646, with macro-averaged specificity, precision, sensitivity, and F1-score values of 0.873, 0.559, 0.557, and 0.548, respectively (Table 2). The class-wise AUC values were 0.901 for Home, 0.874 for Specialized Care, 0.674 for Home with Help, and 0.889 for Expired (Fig 2a, 2b). Robustness assessments indicated excellent model stability and generalization: discrepancies between training and testing accuracy were minimal, differences between test and cross-validation accuracy were less than 4.6%, learning curve analysis showed a negligible rightmost gap (4.7%; Fig 2c), and cross-validation accuracy exhibited low variability (standard deviation = 0.023; Table 2). These findings collectively suggest that the model was stable and generalized well, with a low apparent risk of overfitting or underfitting. In addition, calibration analysis of the final selected MLP classifier demonstrated reasonable, although class-varying, agreement between predicted probabilities and observed event frequencies across the four discharge categories (Fig 2d). The corresponding Brier score / ECE values were 0.130 / 0.060 for home, 0.133 / 0.069 for specialized care, 0.142 / 0.020 for home with help, and 0.048 / 0.018 for expired.

**Table 2:** Comparison of the performance of ten classifiers using overall test-set metrics, cross-validation accuracy, and class-wise AUC values. Cross-validation accuracy ± SD represents the mean cross-validation accuracy with standard deviation across folds. The area under the receiver operating characteristic curve (AUC) 1, 2, 3, and 4 correspond to Home, Specialized Care, Home with Help, and Expired classes, respectively.

Classifier	Test accuracy	Train accuracy	Cross-validation accuracy ± SD	Test Specificity	Test Precision	Test Sensitivity	Test F1-score	AUC 1	AUC 2	AUC 3	AUC 4
**MLP**	**0.646**	**0.662**	**0.600 ± 0.023**	**0.873**	**0.559**	**0.557**	**0.548**	**0.901**	**0.874**	**0.674**	**0.889**
Stacked ensemble (random forest + XGBoost)	0.629	0.665	0.589 ± 0.025	0.865	0.538	0.524	0.531	0.885	0.860	0.660	0.875
XGBoost	0.621	0.680	0.582 ± 0.026	0.862	0.532	0.522	0.527	0.878	0.855	0.655	0.870
Categorical boosting	0.618	0.644	0.572 ± 0.023	0.857	0.524	0.524	0.524	0.862	0.836	0.645	0.851
Random forest	0.610	0.710	0.571 ± 0.028	0.857	0.525	0.515	0.520	0.869	0.846	0.645	0.860
Adaptive boosting	0.602	0.645	0.565 ± 0.022	0.854	0.518	0.512	0.515	0.862	0.838	0.638	0.852
Decision tree	0.589	0.685	0.550 ± 0.035	0.846	0.509	0.499	0.504	0.845	0.820	0.615	0.835
SVC	0.586	0.615	0.548 ± 0.020	0.843	0.500	0.494	0.497	0.840	0.815	0.610	0.830
Logistic regression	0.585	0.605	0.555 ± 0.018	0.840	0.495	0.487	0.491	0.835	0.810	0.605	0.825
KNN	0.517	0.560	0.490 ± 0.025	0.810	0.426	0.420	0.423	0.780	0.750	0.550	0.770

MLP indicates multilayer perceptrons; XGBoost: extreme gradient boosting; SVC: support vector classifier; KNN: K-nearest neighbor.

**Fig 3:**
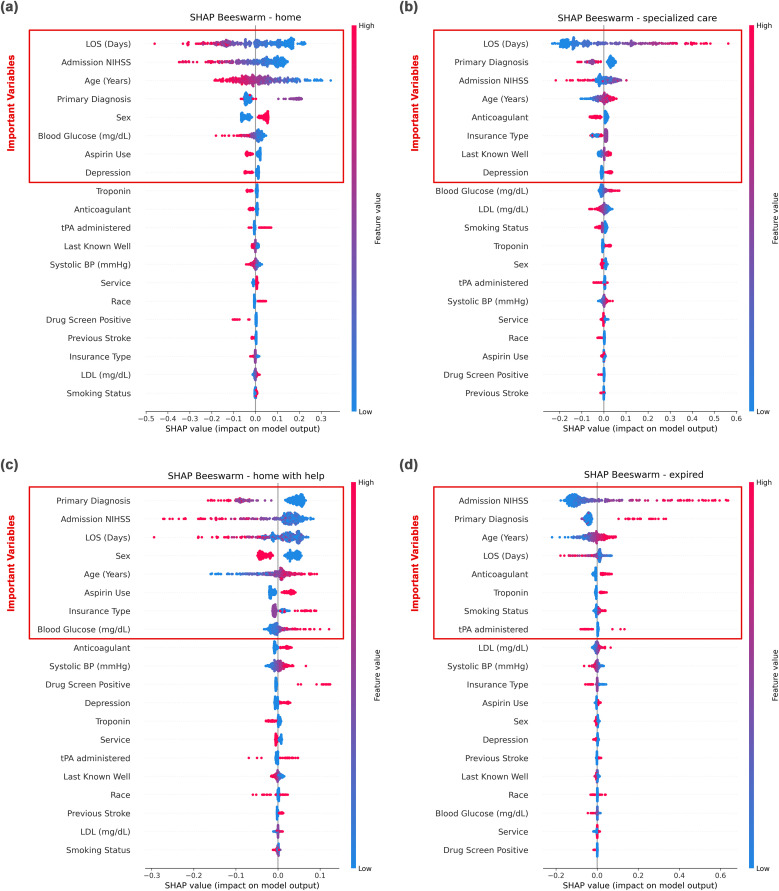
SHapley additive exPlanations (SHAP) summary plots for discharge disposition predictions. Class-specific SHAP values quantify feature contributions to the MLP model’s predictions: (a) Home discharge (Class 0), (b) Specialized care (Class 1), (c) Home with help (Class 2), and (d) Expired (Class 3).

SHAP analysis of the optimized MLP (the best-performing classifier) provided insights into feature contributions for predicting each discharge category. The results indicated that the optimal subset of features comprised the top 80% (S2 Table). Specifically, for predicting discharge to home (class 0), the key predictors by SHAP importance, ranked in descending order, were length of stay (LOS), admission NIHSS, age, primary diagnosis, sex, blood glucose, aspirin use, and depression (Fig 3a). For specialized care (class 1), the key features, ranked by SHAP importance, included LOS, primary diagnosis, admission NIHSS, age, anticoagulant, insurance type, LKW, and depression (Fig 3b). The prediction of home with help (class 2) was most influenced by primary diagnosis, admission NIHSS, LOS, sex, age, aspirin use, insurance type, and blood glucose (Fig 3c). Finally, for expired (class 3), the most critical predictors, ranked by SHAP importance, were admission NIHSS, primary diagnosis, age, LOS, anticoagulant, troponin, smoking status, and tPA administered (Fig 3d). It should be noted that the sensitivity analyses presented in S2 Table confirmed the robustness of the identified key features (Table 2 and S2 Table).

## Discussion

This study introduces an explainable AI framework that predicts stroke discharge disposition across four clinically actionable categories—home, specialized care, home with help, and expired—using demographic, clinical, and treatment-specific variables. The class-specific SHAP patterns provide clinically coherent insight into how the model differentiated discharge destinations. The four predictors shared across all discharge categories—admission NIHSS, LOS, primary diagnosis, and age—represent the central clinical axes of stroke disposition decision-making: neurological injury severity, stroke subtype/pathophysiology, physiologic reserve, and the evolving inpatient trajectory.

**Fig 4:**
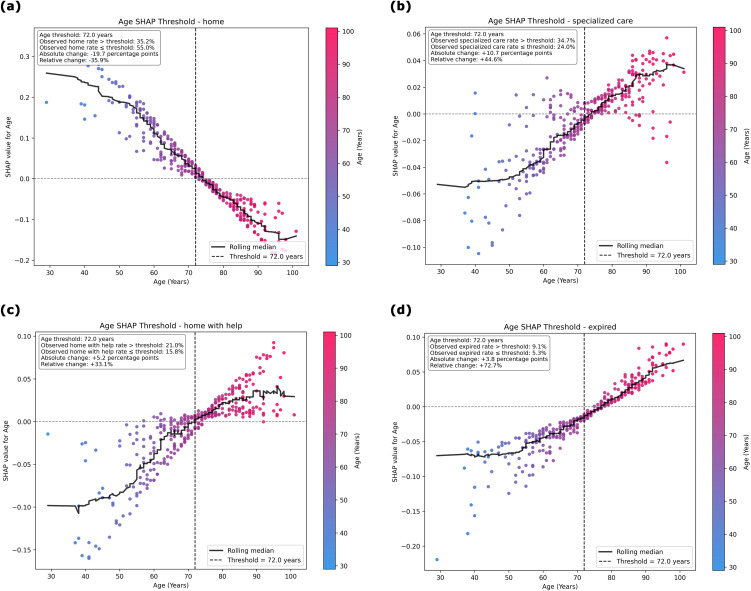
Class-specific SHAP age-threshold analysis for discharge disposition prediction. SHAP dependence plots demonstrate the contribution of age to the final MLP model predictions for each discharge category: (a) Home, (b) Specialized Care, (c) Home with Help, and (d) Expired. Each point represents an individual patient in the hold-out test set, with color indicating age. The black curve represents the rolling median SHAP value, and the vertical dashed line indicates the SHAP-derived age threshold of 72 years. Age above this threshold was associated with a lower likelihood of discharge home and a higher likelihood of discharge to specialized care, discharge home with help, and expired status.

Admission NIHSS is a direct marker of early neurological deficit and therefore strongly informs the likelihood of independent discharge, need for rehabilitation, or mortality. This aligns with the established body of literature recognizing the NIHSS as a robust measure of initial stroke severity and a key predictor of discharge disposition [29]. While some studies have reported weaker associations [19], potentially due to variations in patient populations or methodological limitations, our AI approach effectively addresses the multi-faceted impact of neurological impairment on discharge needs. Furthermore, LOS functions as an integrated marker of clinical complexity, recovery trajectory, inpatient complications, and the time required to coordinate post-acute services. The primary stroke diagnosis also significantly influenced all discharge outcomes. This finding underscores that stroke is a heterogeneous pathophysiological entity, in which the underlying etiology—ischemic, hemorrhagic, or TIA—can influence both the acute clinical course and functional recovery trajectory. Hemorrhagic stroke diagnoses markedly increased the risk of specialized care and mortality, consistent with their inherently severe clinical course and need for comprehensive rehabilitation. It is important to acknowledge that hemorrhagic stroke subtypes, such as subarachnoid hemorrhage, intracerebral hemorrhage, and subdural hematoma, have differing prognoses, and future research should elucidate their specific impact on discharge disposition. Conversely, TIA predominantly led to home discharge, supporting prior research [30]. Age likely reflects baseline frailty, rehabilitation tolerance, comorbidity burden, and social vulnerability, all of which influence discharge planning beyond stroke severity alone. Although some studies have reported no significant association between age and stroke outcomes [18], our findings are consistent with prior work identifying age as an important determinant of clinical outcome [20]. The clinical importance of advanced age has also been emphasized by Torres-Riera et al., who studied 506 women aged ≥85 years with acute ischemic stroke and reported high in-hospital mortality in this subgroup, with sudden stroke onset, altered consciousness, and neurological, cardiac, respiratory, and hemorrhagic complications independently associated with death, whereas lacunar infarction was associated with a favorable prognosis [31]. In the present study, age was included as a continuous predictor, and the SHAP age-threshold analysis identified 72 years as a data-driven threshold for acute stroke discharge disposition prediction (Fig 4). Patients older than 72 years were less likely to be discharged home, with a relative decrease of 35.9%, whereas the relative rates of discharge to specialized care, discharge home with help, and expired status increased by 44.6%, 33.1%, and 72.7%, respectively. These class-specific patterns suggest that increasing age shifts the discharge trajectory away from independent home discharge and toward greater post-acute care needs or mortality, reinforcing the role of age as a contextual marker of vulnerability rather than a purely chronological variable.

The SHAP findings also indicated that tPA administration decreased the likelihood of mortality. This finding is consistent with prior statistical studies demonstrating the benefit of timely tPA administration in improving discharge outcomes [17,32]. In contrast, some statistical analyses and binary-classification AI models have not identified a clear prognostic role for tPA [16,33], potentially because binary endpoints may mask its more nuanced and context-dependent effects across distinct discharge pathways. Although cost considerations remain relevant [17], our findings reinforce the clinical value of tPA in optimizing post-stroke discharge trajectories when used within appropriate clinical indications. Nevertheless, our analysis did not account for tenecteplase, a thrombolytic agent with comparable efficacy demonstrated in trials such as the ATTEST trial and increasingly adopted in contemporary stroke practice [34]. The model also identified anticoagulant medication use as an important predictor for both specialized care and expired discharge categories. This finding should not be interpreted as a direct causal effect of anticoagulation on discharge outcome, but rather as an indicator of the complex clinical context in which anticoagulant use occurs, including cardioembolic risk, atrial fibrillation burden, comorbidity profile, hemorrhagic risk, and the severity of the presenting cerebrovascular event. Prior studies have suggested that anticoagulant therapy may be associated with reduced stroke-related mortality in appropriately selected patients [35,36]. In our model, however, anticoagulant use appeared to contribute to the differentiation of higher-acuity discharge trajectories, particularly specialized care and mortality, likely reflecting the interaction between baseline thromboembolic risk, treatment history, stroke subtype, and acute clinical severity.

Blood glucose and LKW time are particularly important because they represent clinically actionable domains. The association between lower blood glucose levels and greater likelihood of discharge home or home with help suggests that early identification and optimization of dysglycemia may support functional recovery and discharge readiness. Likewise, the contribution of LKW time to specialized care prediction reinforces the clinical impact of delayed presentation and treatment initiation, which may reduce the opportunity for acute intervention and increase rehabilitation needs. Together, these findings highlight modifiable care processes—rapid stroke recognition, timely activation of treatment pathways, and early metabolic optimization—that may improve discharge trajectories after acute stroke. Depression also emerged as a clinically actionable predictor for home discharge and specialized care, emphasizing that discharge disposition is shaped not only by neurological deficit severity but also by neuropsychiatric comorbidity, functional resilience, rehabilitation engagement, social support, and access to structured post-acute resources. This relationship has not been recognized in prior studies constrained by binary outcome frameworks [25,37], which could obscure the more nuanced interaction between mental health and discharge destination. From a clinical standpoint, these findings support early mental health screening and integrated psychosocial assessment during acute stroke care to identify patients at risk for reduced rehabilitation participation, caregiver strain, or discharge delays, thereby improving discharge planning and post-acute care coordination. Aspirin use also contributed to the prediction of both home discharge and discharge home with help, suggesting that antiplatelet therapy may reflect a clinical profile associated with secondary prevention, lower thromboembolic risk, and greater likelihood of functional stabilization sufficient for discharge to a less intensive post-acute care setting.

Sex emerged as an additional important predictor for both home discharge and discharge home with help, whereas insurance type contributed to the prediction of home with help and specialized care. These findings suggest that discharge disposition is influenced not only by neurological severity, but also by socio-behavioral and health-system factors that shape access to post-acute rehabilitation, caregiver support, and home-based services. Prior studies have reported conflicting evidence regarding sex-based differences in post-stroke outcomes and mortality [11,18,19], and our findings suggest that sex may exert a more nuanced influence on discharge pathways rather than acting as a uniform predictor of poor outcomes. Similarly, the contribution of insurance type highlights the potential role of healthcare coverage and resource availability in determining whether patients transition to home-based assistance or structured specialized care.

### Potential clinical implementation of the explainable AI framework

The class-specific SHAP findings highlight the multifactorial nature of discharge disposition after acute stroke and support the clinical plausibility of the model’s predictions. Across all four discharge categories, LOS, admission NIHSS, age, and primary diagnosis emerged as shared key predictors, indicating that the model consistently integrated hospitalization trajectory, neurological injury severity, physiologic reserve, and stroke subtype when estimating discharge destination. Importantly, the SHAP-derived age threshold of 72 years provides a clinically useful marker for early admission-based discharge-risk stratification. Although age itself is not modifiable, identifying patients above this threshold may help clinicians anticipate reduced likelihood of independent home discharge and increased need for specialized care, home-based assistance, or mortality-risk monitoring early in the hospitalization.

Beyond these shared predictors, several SHAP-highlighted variables identify modifiable or addressable care processes. The contribution of blood glucose and LKW time emphasizes the importance of early metabolic optimization, rapid stroke recognition, and timely activation of acute treatment pathways. Similarly, depression highlights the need for early mental health screening and psychosocial assessment, as neuropsychiatric burden may reduce rehabilitation engagement, increase caregiver strain, and delay safe discharge planning. Insurance type, which contributed to the prediction of home with help and specialized care, further underscores that discharge disposition is shaped not only by neurological severity, but also by access to post-acute resources, payer authorization, home health availability, and rehabilitation placement logistics.

In an EHR-integrated workflow, patients predicted to be discharged home with help could trigger early social work involvement, case management, caregiver assessment, home health coordination, and optimization of comorbidities such as dysglycemia or depression. Patients predicted to require specialized care could prompt earlier rehabilitation consultation, therapy planning, insurance authorization, and communication with post-acute facilities. Patients at elevated risk for expired status could prompt intensified neurological stabilization, cardiopulmonary monitoring, and goals-of-care discussions when clinically appropriate. Thus, the proposed explainable AI framework may support earlier and more individualized care planning by linking predicted discharge trajectories to transparent SHAP-based explanations, while complementing rather than replacing clinician judgment.

### Limitations and future directions

This single-center retrospective study has several limitations. Although the dataset included routinely available clinical variables, it lacked more granular predictors, such as detailed neuroimaging characteristics, precise infarct or hemorrhage volume, lesion location, vascular territory involvement, edema burden, and markers of mass effect or secondary hydrocephalus. Prior computational studies relating cerebrospinal fluid hydrodynamic alterations to clinical symptoms and treatment response in neurosurgical diseases have demonstrated the prognostic value of patient-specific biomechanical data [38–40]. Extending this paradigm to stroke discharge disposition, future AI frameworks could integrate advanced numerical and fluid–structure interaction markers to capture the mechanical impact of stroke-related edema, hemorrhage, or secondary hydrocephalus. In addition, the complex and context-dependent role of anticoagulant use warrants targeted investigation in specific clinical settings such as atrial fibrillation management, and the prognostic implications of specific hemorrhagic stroke subtypes (e.g., subarachnoid, intracerebral, and subdural hemorrhage) were not analyzed separately, limiting our understanding of their distinct effects on discharge disposition. Moreover, the focus on acute hospitalization data did not capture factors such as ongoing recovery potential and post-discharge rehabilitation quality, which are important determinants of longer-term outcomes and should be incorporated into future longitudinal models. Heterogeneity across acute stroke categories, including etiologic ischemic stroke subtypes such as cardioembolic, lacunar, atherothrombotic, unusual-etiology, and undetermined-etiology infarction, may also have influenced outcome prediction but was not explicitly modeled in the present study. Finally, although the current AI framework demonstrated favorable internal performance, it should still be regarded as a feasibility proof-of-concept. External validation across multiple institutions is required before development of a clinical prototype or implementation in bedside decision-making. Potential effects on discharge efficiency, resource allocation, and health-system outcomes should be evaluated prospectively before clinical deployment.

## Conclusion

This study demonstrates the feasibility of an explainable AI framework for multiclass prediction of discharge disposition after acute stroke using routinely available EHR variables. The final MLP model identified clinically plausible and class-specific predictors of discharge to home, specialized care, home with help, and expired status. SHAP interpretation showed that admission NIHSS, LOS, age, and primary diagnosis were shared predictors across discharge categories, while modifiable or addressable factors such as blood glucose, LKW time, depression, insurance type, anticoagulant use, troponin status, and tPA administration provided additional class-specific insight. The SHAP-derived age threshold of 72 years further supported early admission-based discharge-risk stratification. Integration of this framework into EHR-based emergency workflows may support timely alerts for patients at risk of requiring specialized care, home assistance, or mortality-focused care pathways. Such alerts may facilitate earlier rehabilitation consultation, social work involvement, home health referral, glycemic optimization, depression screening, and goals-of-care discussions when appropriate. From a health-system perspective, this approach may improve allocation of rehabilitation beds, coordination of post-acute services, and efficiency of care transitions. Future research should focus on external multicenter validation, age- and subtype-specific modeling, and prospective evaluation of EHR-integrated decision-support tools that dynamically update predictions across the care continuum. Future studies should also assess whether such tools can improve discharge planning, rehabilitation allocation, and broader health-system efficiency.

## Supporting information

S1 Fig Clinical protocol for suspected stroke management, including neurological assessment, emergent CT imaging, thrombolytic eligibility evaluation, and disposition pathways. ED: Emergency Department; ABCs: Airway, Breathing, Circulation; IV: Intravenous; NIH score: National Institutes of Health Stroke Scale; CT scan: Computed Tomography scan; ECG: Electrocardiogram; PO/PR: Per Os / Per Rectum (PO = by mouth, PR = by rectum); ICU: Intensive Care Unit.
(JPG)

S1 Table Hyperparameter search spaces and final selected settings for each classifier using stratified 5-fold cross-validation. (DOCX)

S2 Table Multilayer perceptron (MLP) model performance metrics across SHapley Additive exPlanations (SHAP)-derived feature subsets (65–85% thresholds) with feature perturbations. Cross-validation accuracy ± SD: Mean cross-validation accuracy with standard deviation across folds. Learning curve gap (%): Difference (%) between training and cross-validation accuracy at maximum training size. MLP: Multilayer Perceptron; SHAP: SHapley Additive exPlanations; SD: Standard deviation; AUC: Area under the ROC curve
(DOCX)

S1 Checklist TRIPOD+AI checklist for reporting the development and evaluation of the explainable artificial intelligence model. (PDF)

S1 Code Supplementary analysis code and serialized model-related artifacts generated during the current study. The archived release corresponding to this manuscript is available through Zenodo at: https://doi.org/10.5281/zenodo.20711230. The GitHub repository is available at: https://github.com/Seif-AI-Lab/Ai-Stroke-Discharge-PLOS-One.
(ZIP)

## Abbreviations

AI: Artificial Intelligence
NIHSS: NIH Stroke Scale
tPA: tissue plasminogen activator
SHAP: SHapley Additive exPlanations
mRS: modified Rankin Scale
BI: Barthel Index
IRB: Institutional Review Board
EHR: electronic health records
TIA: transient ischemic attack
ED: emergency department
LDL: low-density lipoprotein
MICE: Multiple Imputation by Chained Equations
ANOVA: analysis of variance
SVC: support vector classifier
KNN: k-nearest neighbors
XGBoost: extreme gradient boosting
MLP: multilayer perceptron
SMOTE: Synthetic Minority Over-sampling Technique
AUC: area under the receiver operating characteristic curve
PCA: principal component analysis
ECE: expected calibration error
LOS: length of stay
LKW: last known well
AUROC: area under the receiver operating characteristic curve
OvO: one-vs-one
OvR: one-vs-rest
ECDF: empirical cumulative distribution function
BP: blood pressure
