# Supplementary figures and images for: An explainable artificial intelligence framework for clinical decision support in stroke discharge planning
Source: PLoS One. 2026 Jul 15;21(7):e0353683. doi: 10.1371/journal.pone.0353683 (PMC13372143; doi:10.1371/journal.pone.0353683)

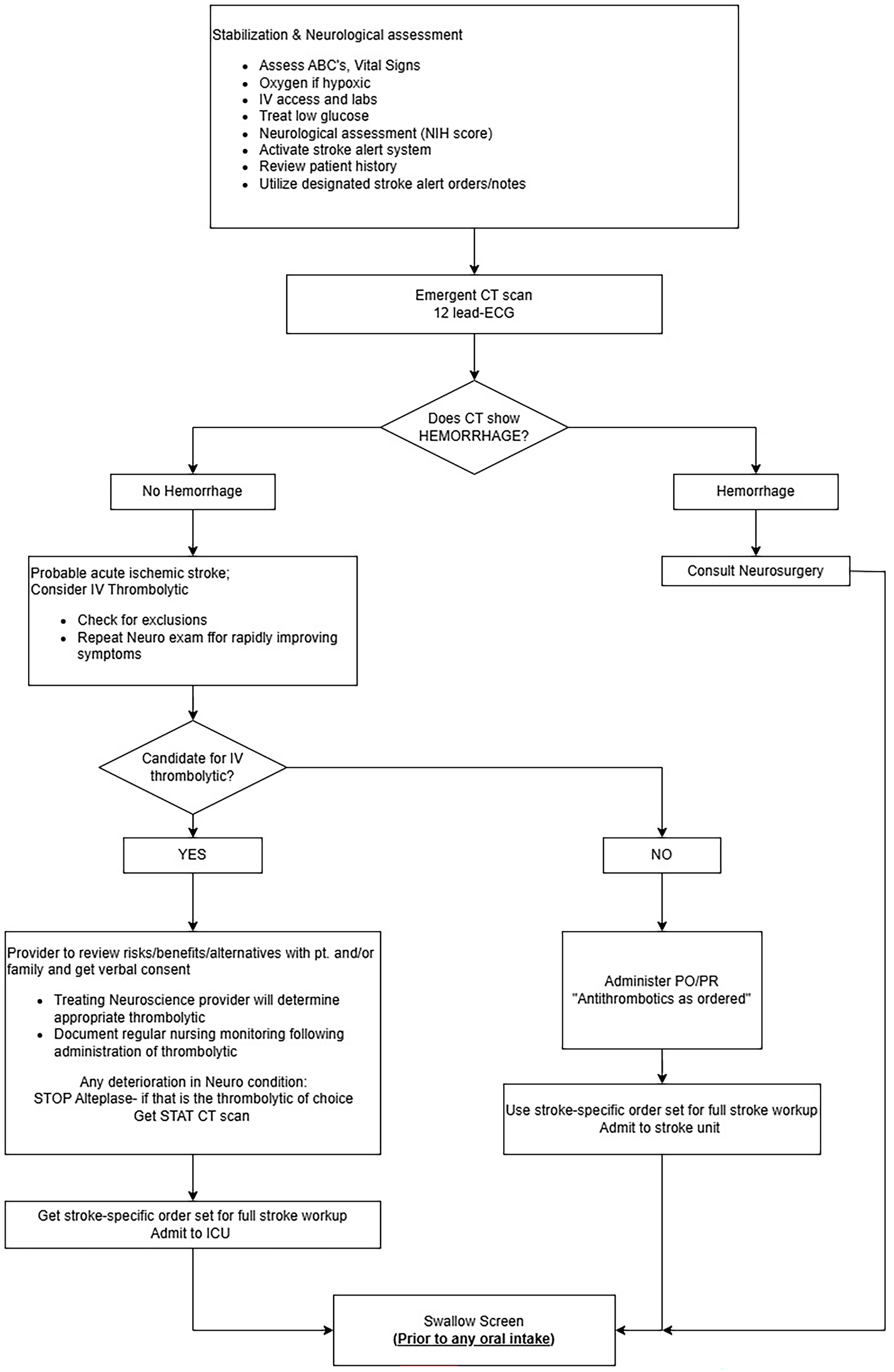

Supplement: S1 Fig — ED: Emergency Department; ABCs: Airway, Breathing, Circulation; IV: Intravenous; NIH score: National Institutes of Health Stroke Scale; CT scan: Computed Tomography scan; ECG: Electrocardiogram; PO/PR: Per Os / Per Rectum (PO = by mouth, PR = by rectum); ICU: Intensive Care Unit. (JPG) [file pone.0353683.s001.jpg]
