# Supplementary material for: An explainable artificial intelligence framework for clinical decision support in stroke discharge planning
Source: PLoS One. 2026 Jul 15;21(7):e0353683. doi: 10.1371/journal.pone.0353683 (PMC13372143; doi:10.1371/journal.pone.0353683)
Supplement: S1 Table — (DOCX) [file pone.0353683.s002.docx]

**S1 Table.** Hyperparameter search spaces and final selected settings for each classifier using stratified 5-fold cross-validation.

| **Classifier** | **Hyperparameters and Grid Search Space** |
| --- | --- |
| **Multilayer Perceptron (MLP)** | hidden_layer_sizes = (48,), activation = logistic, learning_rate = constant, learning_rate_init = 0.0007, batch_size = 8, max_iter = 3000, tol = 0.0001, alpha = 0.03, early_stopping = True, validation_fraction = 0.25, n_iter_no_change = 35, class_weight_multiplier = 1.0, majority_weight_divisor = 1.0, and resample_size_multiplier = 2.5 |
| **Stacked Ensemble (Random forest + XGBoost)** | Base learners consisted of Random Forest and XGBoost using their independently optimized hyperparameters. The meta-learner was Logistic Regression, with meta_learner__C: [0.1, 1.0, 10.0] |
| **XGBoost** | n_estimators: [100, 200, 300]max_depth: [3, 5, 7]learning_rate: [0.01, 0.05, 0.1]subsample: [0.8, 0.9, 1.0]colsample_bytree: [0.8, 0.9, 1.0] |
| **Categorical Boosting (CatBoost)** | iterations: [100, 200, 300]depth: [4, 6, 8]learning_rate: [0.01, 0.05, 0.1]l2_leaf_reg: [1, 3, 5]loss_function: ['MultiClass'] |
| **Random Forest** | n_estimators: [100, 200, 300]max_depth: [None, 10, 20]min_samples_split: [2, 5, 10]min_samples_leaf: [1, 2, 4]class_weight: ['balanced', 'balanced_subsample'] |
| **Adaptive Boosting (AdaBoost)** | n_estimators: [50, 100, 200]learning_rate: [0.01, 0.1, 0.5, 1.0]algorithm: ['SAMME', 'SAMME.R'] |
| **Decision Tree** | criterion: ['gini', 'entropy']max_depth: [None, 5, 10, 15, 20]min_samples_split: [2, 5, 10]min_samples_leaf: [1, 2, 4]class_weight: ['balanced', None] |
| **Support Vector Classifier (SVC)** | C: [0.1, 1, 10, 100]kernel: ['linear', 'rbf']gamma: ['scale', 'auto', 0.001, 0.01]class_weight: ['balanced', None] |
| **Logistic Regression** | penalty: ['l2']C: [0.01, 0.1, 1.0, 10, 100]solver: ['lbfgs']class_weight: ['balanced', None] |
| **K-Nearest Neighbors (KNN)** | n_neighbors: [3, 5, 7, 9, 11]weights: ['uniform', 'distance']metric: ['euclidean', 'manhattan'] |
