# Supplementary material for: An explainable artificial intelligence framework for clinical decision support in stroke discharge planning
Source: PLoS One. 2026 Jul 15;21(7):e0353683. doi: 10.1371/journal.pone.0353683 (PMC13372143; doi:10.1371/journal.pone.0353683)
Supplement: S2 Table — Cross-validation accuracy ± SD: Mean cross-validation accuracy with standard deviation across folds. Learning curve gap (%): Difference (%) between training and cross-validation accuracy at maximum training size. MLP: Multilayer Perceptron; SHAP: SHapley Additive exPlanations; SD: Standard deviation; AUC: Area under the ROC curve (DOCX) [file pone.0353683.s003.docx]

**S2 Table.** Multilayer perceptron (MLP) model performance metrics across SHapley Additive exPlanations (SHAP)-derived feature subsets (65–85% thresholds) with feature perturbations. The area under the receiver operating characteristic curve (AUC) 1, 2, 3, and 4 correspond to Home, Specialized Care, Home with Help, and Expired classes, respectively.

| **Features** | **Test Accuracy** | **Test Specificity** | **Test Precision** | **Test Sensitivity** | **Test**  **F1-score** | **AUC 1** | **AUC 2** | **AUC 3** | **AUC 4** |
| --- | --- | --- | --- | --- | --- | --- | --- | --- | --- |
| Aggregated four discharge categories top features with 65% of SHAP value | 0.581 | 0.835 | 0.503 | 0.501 | 0.502 | 0.855 | 0.828 | 0.628 | 0.843 |
| Aggregated four discharge categories top features with 70% of SHAP value | 0.603 | 0.848 | 0.518 | 0.516 | 0.517 | 0.870 | 0.843 | 0.643 | 0.858 |
| Aggregated four discharge categories top features with 75% of SHAP value | 0.627 | 0.862 | 0.538 | 0.536 | 0.537 | 0.890 | 0.863 | 0.663 | 0.878 |
| Aggregated four discharge categories top features with 80% of SHAP value + 1 feature | 0.642 | 0.870 | 0.546 | 0.544 | 0.545 | 0.898 | 0.871 | 0.671 | 0.886 |
| **Aggregated four discharge categories top features with 80% of SHAP value** | **0.646** | **0.873** | **0.559** | **0.557** | **0.548** | **0.901** | **0.874** | **0.674** | **0.889** |
| Aggregated four discharge categories top features with 80% of SHAP value - 1 feature | 0.649 | 0.875 | 0.552 | 0.550 | 0.551 | 0.904 | 0.877 | 0.677 | 0.892 |
| Aggregated four discharge categories top features with 85% of SHAP value | 0.654 | 0.878 | 0.558 | 0.556 | 0.557 | 0.910 | 0.883 | 0.683 | 0.898 |

AUC: area under the receiver operating characteristic curve; SHAP: SHapley Additive exPlanations.
